# Energy-Efficient Forest Fire Prediction Model Based on Two-Stage Adaptive Duty-Cycled Hybrid X-MAC Protocol

**DOI:** 10.3390/s18092960

**Published:** 2018-09-05

**Authors:** Jin-Gu Kang, Dong-Woo Lim, Jin-Woo Jung

**Affiliations:** Department of Computer Science and Engineering, Dongguk University, Seoul 04620, Korea; kanggu12@dongguk.edu (J.-G.K.); aehddn@gmail.com (D.-W.L.)

**Keywords:** forest fire, prediction model, energy efficient, sensors, Wireless Sensor Network, X-MAC, hybrid, adaptive, duty-cycle, protocol

## Abstract

This paper proposes an adaptive duty-cycled hybrid X-MAC (ADX-MAC) protocol for energy-efficient forest fire prediction. The Asynchronous sensor network protocol, X-MAC protocol, acquires additional environmental status details from each forest fire monitoring sensor for a given period, and then changes the duty-cycle sleep interval to efficiently calculate forest fire occurrence risk according to the environment. Performance was verified experimentally, and the proposed ADX-MAC protocol improved throughput by 19% and was 24% more energy efficient compared to the X-MAC protocol. The duty-cycle was shortened as forest fire probability increased, ensuring forest fires were detected at faster cycle rate.

## 1. Introduction

Forest fires spread rapidly according to topography and mountain slope if they cannot evolve at an early stage due to their nature. In particular, forest fires generated during the day spread more widely, cannot evolve, and are transferred to deeper mountain areas.

**Figure 1:**
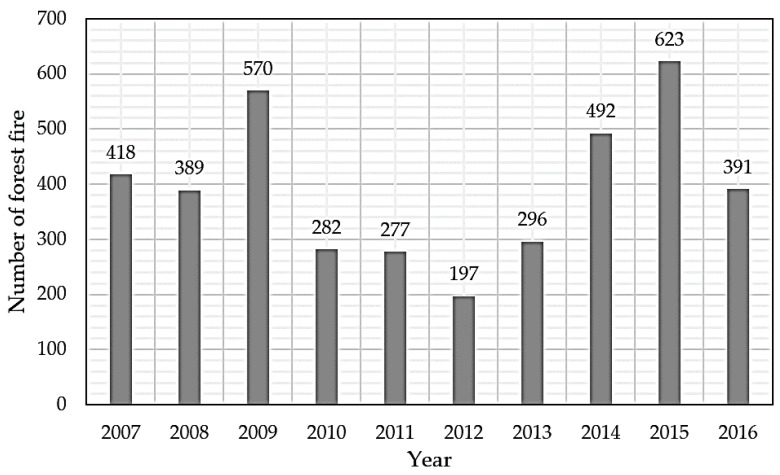
South Korea total yearly forest fires.

Figure 1 shows forest fire occurrence in South Korea. An average of 340,000 people die every year due to forest fires worldwide [1], and average yearly damage caused by forest fires over the last 10 years was approximately 10 million US Dollars [2]. Destroyed forests, damaged buildings, and human injuries and casualties due to forest fires should all be avoided. Forest fire watching, administered separately by local governments in South Korea, has a human cost of approximately 0.5 million US Dollar for each local government [1]. The goal of this paper was to reduce this damage, recovery, and maintenance costs without increasing forest fire risk. 

The average forest area in South Korea is about 6 million hectares [2]. When the proposed protocol is applied to a sensor capable of covering a 300 m range, the number of required sensors is about 1000. It does not cover all areas of the forest but is the central area and the passenger path where forest fires can intensify. The areas that are actually in contact with the surface and the summits are very unlikely to develop into forest fires, and the fire is naturally turned off. When the price of one sensor is set at 200 US Dollar, it costs about 2.2 million US Dollar for maintenance costs, such as sensor cost and battery replacement. The cost of the sensor using the protocol proposed in this paper is about 1/4 times lower than the existing cost, and the 24 h surveillance is cost effective. There is an advantage in that it is possible. The biggest advantage of being able to keep watch 24 h is that you can completely prevent forest fires. In addition, even if the existing labor cost is replaced with a sensor, the maintenance cost of the battery also exists. When sensors are used, energy-efficiency is the most considered part.

Synchronous and asynchronous medium access control (MAC) protocols have been proposed to reduce energy consumption, e.g., such as idle energy [3,4], which operates on the basis of a duty-cycle. Traffic increases as the number of connected devices increases, but node duty-cycles remain unchanged. Consequently, opportunities to send data decrease and throughput decreases, as listening interval durations increase. In particular, a forest fire’s sudden and natural escalation means there is small traffic by the initial sensors that very rapidly increases as the fire develops.

Therefore, an adaptive duty-cycled hybrid X-MAC (ADX-MAC) protocol based on X-MAC protocol [5] is proposed to implement an energy efficient forest fire prediction system that more efficiently reduces energy consumption during sensor waiting times and speeds up data transmission between nodes.

## 2. Forest Fire Prediction

The forest fire prediction model [6] is selected by data used, including nationwide forest fire data for 25 years (1991–2015), consisting of 10,560 observations and other details regarding forest fire areas and characteristics. Table 1 summarizes the variables included in the forest fire damage records [1,6,7].

### 2.1. Prediction Model Weights

The prediction model used the weather and location information relevant to the forest fire occurrence and parameters for human accessibility. It employed spatial kriging to include meteorological information, distance from dense populations to the fire location, number of trails in the lattice, and altitude data to generate accessibility parameters. The available data was modified to correspond to a 5 km grid. The experiment selected logistic regression as the forest fire prediction model and it was reflected in the monthly effect model using variable numbers for refinement. The model achieved 84.8% predicted rate, which was very significant.

Main forest fire contributing factors include temperature, wind speed, humidity, and rainfall. Table 2 shows the correlations for these climate factors according to the forest fire prediction model.

The correlation between the number of forest fires and temperature (*TP*) is almost insignificant, whereas wind speed (WS), humidity (H), and rainfall (R) correlations are all significant at this level. Thus, forest fire probability increases when wind speed is high, humidity is low, and rainfall is small.

Forest fire risk in terms of weighted climate factors can be estimated as:

(1) $$\;f_{\mathrm{risk}}\left(t\right)=\left|\frac{\Delta_{WS}\left(t\right)}{\Delta_{TP}\left(t\right)\Delta_{H}\left(t\right)\Delta_{R}\left(t\right)}\right|,\;$$

where

(2) $$\Delta_{X}(t)=\{\begin{array}{cc} w_{X}\frac{X(t)\;-\;X_{\min}}{X_{\max}\;-\;X_{\min}}, & \;\;X(t)>X_{\min}\\ 1, & \;\;X(t)\leq X_{\min} \end{array}$$

where $X_{t}$ is the measured climate factor over time frame *t* with $X_{max}$ and $X_{\min}$ maximum and minimum, respectively, over the period; and $w_{X}$ is the correlation coefficient from Table 1, i.e., the weight for each factor. Therefore, each $\Delta_{X}$ is a weighted ratio to the uniform average change of all factors.

### 2.2. Forest Fire Risk Analysis from Test Statistics and Weights 

**Figure 2:**
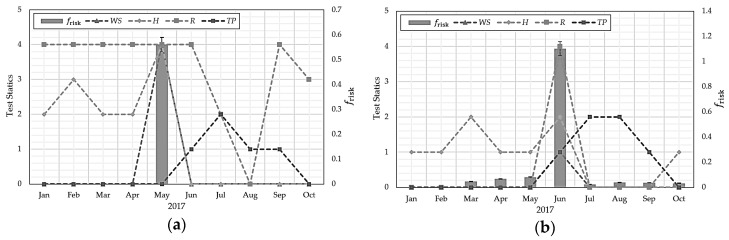
Test statistic variation with duty-cycle length for (**a**) Mt. Gangneung and (**b**) Mt. Surak.

Table 3 and Table 4 show climate factors for Gangneung and Surak Mountains in South Korea for January to October 2017 [2,7]. Stair function test statistics included 0–5, considering the minimum and maximum for each climate factor in the mountain range. Figure 2 shows the data from Table 3 and Table 4 graphically.

The stair function is to compute each climatic factor as a single graph regardless of the unit dimension, and it is divided into stages according to the numerical value of each climatic factor. WS is more than 5 to 11 m/s, H is 60 to 30%, R is 400 to 100 mm, and *TP* is 20 to 35 °C. The figures set for each step show the average degree of climate change in the forest area of South Korea.

The Mt. Gangneung forest fire, 8 May 2017, had $f_{\mathrm{risk}}$ = 0.5610, i.e., very high compared to normal values, and the Mt. Surak forest fire, 1 June 2017, had $f_{\mathrm{risk}}$ = 1.102, i.e., extremely high. Thus, model estimates are reasonable, and hence $f_{\mathrm{risk}}$ was adopted as a core parameter for the proposed protocol. However, $f_{\mathrm{risk}}$ does not specify that a forest fire will occur, but only that the probability of occurrence is high. Figure 2 shows that $f_{\mathrm{risk}}$ is most influenced by WS among the climate factors, but there are some factors considered by other climate factors.

## 3. Energy Efficient Forest Fire Prediction Protocol

### 3.1. Duty-cycle Wireless Sensor Networks

**Figure 3:**
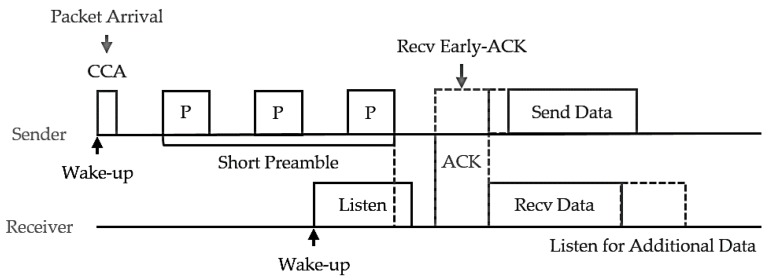
X-MAC protocol.

Figure 3 shows the asynchronous sensor network protocol, i.e., the X-MAC protocol [4], that was proposed to solve the Berkley MAC (B-MAC) low power listening preamble overhearing problem [8]. X-MAC uses the basic B-MAC methods, but in contrast to the long B-MAC preamble, it transmits the node address as a short preamble followed by a listening to ensure they will receive it.

A receiver node receives the preamble containing its own address value and sends an Early-ACK to a sender node to inform it of preamble reception. When the sender node receives the Early-ACK, it stops transmitting the preamble and immediately transmits the data, thereby transferring data between the two nodes. If the receiver node does not detect its address in the preamble, it immediately returns to idle mode. Thus, control packet overhead is significantly reduced compared to B-MAC, and returning to idle mode effectively reduces overhearing.

**Figure 4:**
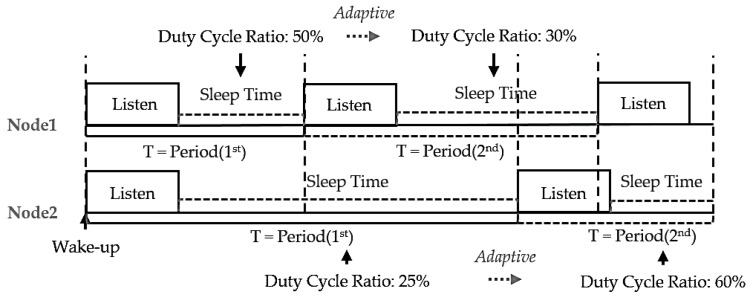
Proposed adaptive duty-cycled hybrid X-MAC protocol.

Figure 4 shows the proposed adaptive duty-cycled hybrid X-MAC (ADX-MAC) protocol [9].

Normal X-MAC has a constant duty-cycle, whereas the proposed ADX-MAC duty-cycle, and hence sleep interval, changes according to the current situation. This has a similar effect to contention window (CW) [10] for preventing collision, hence collision probability within a given time (slot) is significantly reduced because the node duty is staggered without Backoff [11], reducing unnecessary delay. For example, when the number of packets in each node’s queue is less than the number of packets in the queue, the sleep cycle is increased by decreasing the duty-cycle, reducing energy consumption. On the other hand, when the number of packets increases, particularly when the queue maximum is reached, the duty-cycle is increased, but as little as possible, shortening the sleep interval, and traffic can be quickly resolved. Adapting the duty-cycle to the traffic situation improves energy efficiency compared to throughput by adjusting the unnecessary sleep interval and the time when the node wakes up.

### 3.2. Proposed Adaptive Duty-cycled Hybrid X-MAC

The proposed ADX-MAC condition for controlling duty-cycle length is the number of packets in the queue and $f_{\mathrm{risk}}$ value.

**Figure 5:**
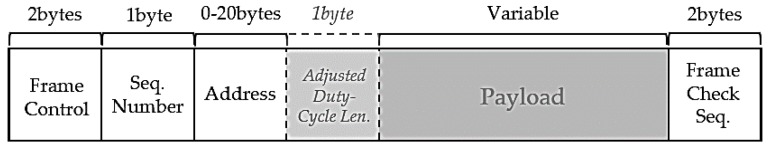
MAC Header (Data Unit) of ADX-MAC.

Figure 5 shows that the proposed ADX-MAC header components are almost identical to the X-MAC. However, when the sender sends data to the receiver and the receiver and sender have a similar cycle, the sender’s duty-cycle length is included in the header content and may also be included in the Early-ACK or ACK transmission, in contrast to the usual MAC header.

**Figure 6:**
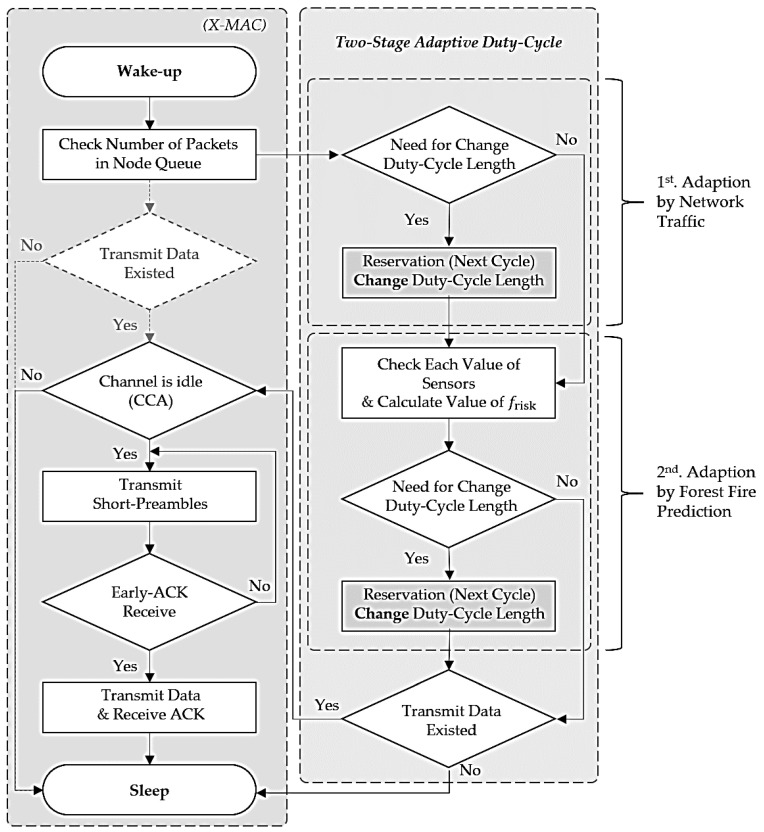
Proposed ADX-MAC algorithm for forest fire.

Figure 6 shows the proposed ADX-MAC algorithm. When the sender wakes up, it first checks the node queue status too determine if there is data to be sent, and if so, operates identically to the X-MAC algorithm, except that X-MAC checks channel status immediately, whereas ADX-MAC first checks duty-cycle length to determine if change (adjust) is required. If duty-cycle length needs to change, this is scheduled (reserved) for the next cycle. If no change is required, the process proceeds to the next step.

The ratio of the number of packets in the queue to the total maximum queue length determines the duty-cycle length for the queue:

(3) $$Q_{\mathrm{ratio}}\left(t\right)\;=\;Q\left(t\right)/Q_{\max},$$

where *Q*(*t*) is the number of packets queued at current time, *t*; and $Q_{\max}$ is the maximum number of packets that can be queued.

It is then decided whether to change duty-cycle length according to $f_{\mathrm{risk}}$. If there is data to send immediately, then any duty-cycle change is scheduled for the next cycle. If no change is required the node checks channel status and then transmits the short preamble until it receives Early-ACK. After the data is transmitted the node switches to sleep state.

The increase rate per unit time is:

(4) $$f_{\mathrm{incr}}\left(t\right)=\left\{ \begin{array}{cc} f_{\mathrm{risk}}\left(t\right)/f_{\mathrm{risk}}\left(t_{0}\right), & \;\;f_{\mathrm{risk}}\left(t_{0}\right)\neq 0\\ 1, & \;\;f_{\mathrm{risk}}\left(t_{0}\right)=0\\ 0, & \;\;t=0 \end{array}\right.$$

where $t_{0}$ is the previous time unit and *t* is the current time unit.

This is divided into two stages because forest fires develop extremely quickly, and traffic congestion can occur during a one-time unit. Therefore, *Q*(*t*) measure channel congestion and $f_{\mathrm{risk}}$ predict and respond quickly to the situation before it occurs, sending emergency data without interruption as the forest fire develops. Thus, combining Equations (3) and (4), the new adaptive duty-cycle is:

(5) $$\;T_{\mathrm{adp}}(t)=\{\begin{array}{cc} 1-\left(f_{\mathrm{incr}}\left(t\right)+Q_{\mathrm{ratio}}\left(t\right)\right)+f_{\mathrm{incr}}\left(t\right)Q_{\mathrm{ratio}}\left(t\right), & \;\;f_{\mathrm{incr}}\left(t\right)\leq f_{\mathrm{thd}}\\ 1-\left(f_{\mathrm{thd}}+Q_{\mathrm{ratio}}\left(t\right)\right)+f_{\mathrm{thd}}Q_{\mathrm{ratio}}\left(t\right), & \;\;f_{\mathrm{incr}}\left(t\right)>f_{\mathrm{thd}} \end{array}\;$$

where $f_{\mathrm{thd}}$ is an arbitrary threshold to limit $f_{\mathrm{incr}}$. Thus, the applied duty-cycle length is:

(6) $$\;T\left(t\right)=\{\begin{array}{cc} T_{\mathrm{adp}}\left(t\right)T_{0}, & \;\;T_{\mathrm{adp}}\left(t\right)T_{0}\geq T_{\mathrm{listen}}\\ T_{\mathrm{listen}}, & \;\;T_{\mathrm{adp}}\left(t\right)T_{0}<T_{\mathrm{listen}} \end{array}\;$$

which can grow very large for severe climate factor changes. Although *T* depends on values obtained from Equations (3) and (4), T cannot be smaller than the listen time, $T_{\mathrm{listen}}$, if calculated *T* < $T_{\mathrm{listen}}$, $T\;\to T_{\mathrm{listen}}$, and hence duty-cycle ratio = 100%.

## 4. Experimental Results

The first experiment compares the throughput and energy consumption with X-MAC to confirm the performance of the proposed protocol, ADX-MAC. However, the number of nodes in this experiment is limited to 5 to 60. The reason for this is that, since the characteristics of the sensor MAC protocol, the collision rate is close to 99% as long as the CW is not significantly larger than 60 since the performance is significantly lowered. That is, it is the same as the case where all the nodes simultaneously send data during the unit interval in the saturation situation. Therefore, it is possible to confirm the difference in performance between two protocols in a real environment even with this experiment alone. The simulator used in this experiment was a proven NS-2 based simulator [12,13].

Verification in the actual forest fire situation is the most accurate, but it is difficult to generate forest fires artificially. The condition of spontaneous ignition is due to the combination of various climatic factors. Of course, social issues are also included. Therefore, in the second experiment for the forest fire prediction model, each climate factor was measured through an Arduino sensor for a certain unit time. And we confirmed that these measured values matched the duty-cycle length properly.

### 4.1. Comparison with X-MAC

Table 5 shows the simulation environment employed. The simulation assumed the same environment and topography for both models, in particular, node distances were the same, packet generation used the constant bit rate method, and the network was always unsaturated.

Throughput was calculated as

(7) $$\;THR=\frac{C_{\mathrm{ack}}PKT_{\mathrm{size}}}{t_{\mathrm{stop}}-t_{\mathrm{start}}},\;$$

where $C_{\mathrm{ack}}$ is the number of ACKs received by the sender until the end of the simulation; $PKT_{\mathrm{size}}$ is the packet size; and $t_{\mathrm{stop}}$ and $t_{\mathrm{start}}$ indicate the time the simulation ended and started, respectively.

Energy consumption was calculated as:

(8) $$\;E=\frac{PWR_{\mathrm{use}}}{N\left(t_{\mathrm{stop}}-t_{\mathrm{start}}\right)}\;$$

where $PWR_{\mathrm{use}}$ is the power used until the end of the simulation, and N is the total number of nodes.

**Figure 7:**
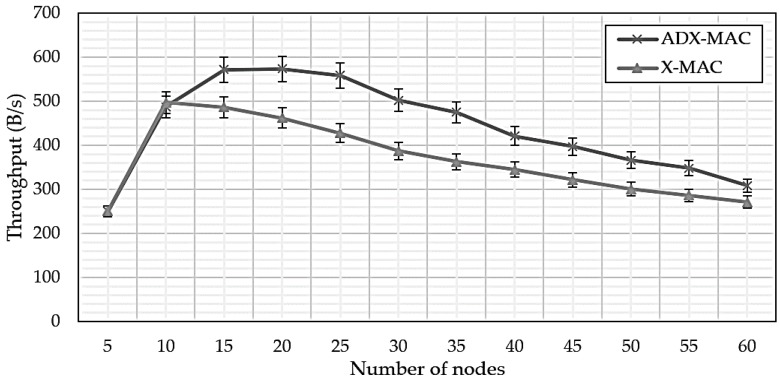
ADX-MAC and X-MAC throughput.

This experiment did not consider $f_{\mathrm{risk}}$, because there would be little throughput effect if this was included. Figure 7 compares ADX-MAC and X-MAC throughput. Efficiency is similar up to nodes = 10, but ADX-MAC has an average efficiency approximately 24% higher than X-MAC once nodes = 15, and this advantage persists up to quite large networks.

**Figure 8:**
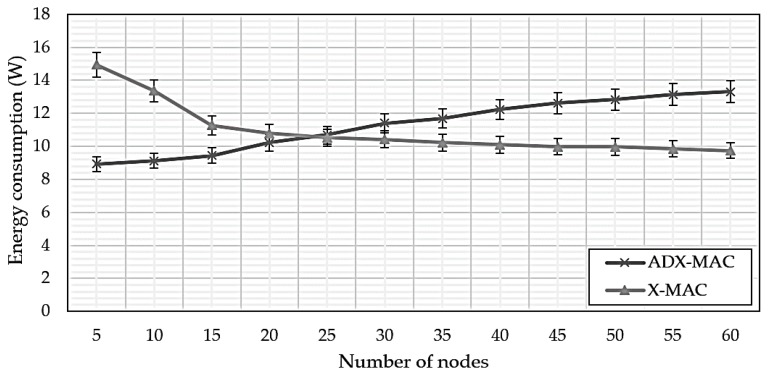
ADX-MAC and X-MAC energy consumption.

Figure 8 compares ADX-MAC and X-MAC energy consumption. Average ADX-MAC efficiency was approximately 18% lower than that of X-MAC up to nodes = 25, which then extended to 24% lower. This result is due to the fact that ADX-MAC performs transmission about 1/4 the rate of X-MAC as the number of nodes increases.

**Figure 9:**
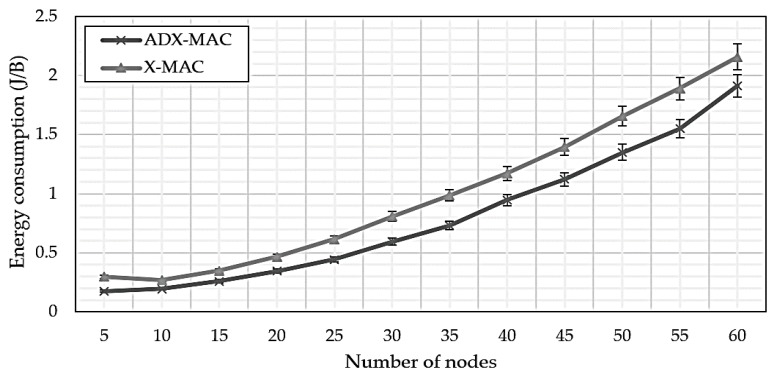
ADX-MAC and X-MAC energy consumption for an individual byte transmitted until the simulation completed.

Figure 9 compares ADX-MAC and X-MAC energy consumption per byte, i.e., the average amount of energy consumed to transmit one byte until the simulation ended, calculated as:

(9) $$\;E_{\mathrm{byte}}=\frac{PWR_{\mathrm{use}}}{C_{\mathrm{ack}}PKT_{\mathrm{size}}}.\;$$

The amount of energy consumed per byte can be said to be always good because the ADX-MAC consumes less than the X-MAC over the number of intervals of all nodes. Although the efficiency of ADX-MAC is about 28% higher than that of X-MAC until the number of nodes is 35, the efficiency drops rapidly after that. However, the average number of nodes is about 17% in the range of 40 to 60. In conclusion, it shows about 24% efficiency in the all number of nodes.

### 4.2. Adaptive Duty-Cycle Length Due to Climatic Factors

This experiment investigated how *T* varied with measured climate factor values. Network setup and parameters were the same as in Section 4.1, but this experiment did not consider the state of the queue, similar to the treatment for $f_{\mathrm{risk}}$ in Section 4.1.

**Figure 10:**
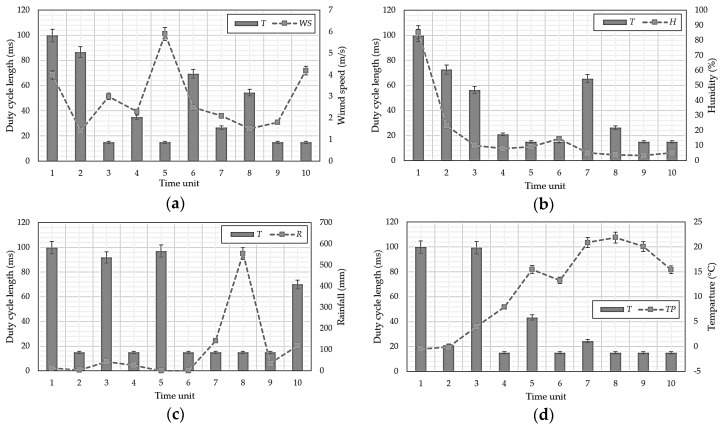
Duty-cycle length, *T*, changes for climate factor changes (**a**) wind speed; (**b**) humidity; (**c**) rainfall, and (**d**) temperature.

**Figure 11:**
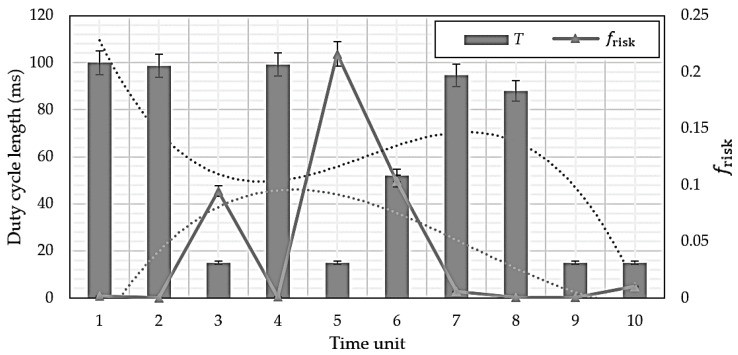
Change in $f_{\mathrm{risk}}$ as duty-cycle length, *T*, changes.

Figure 10 shows the *T* changes for each climate factor, considering only the factor of interest to be changing, whereas Figure 11 considers all climatic factors. High $f_{\mathrm{risk}}$ derived from the forest fire prediction model means forest fire probability is high, hence *T* is shortened, and sensing is performed at a shorter cycle per unit time.

## 5. Conclusions

This paper proposed a forest fire prediction method, i.e., the adaptive duty-cycled hybrid X-MAC (ADX-MAC) protocol, which is based on sensor data to reduce human and economic costs using Wireless Sensor Network (WSN) MAC protocol. The key factors of the proposed method were to adapt duty-cycle length through two stages with different factors. The two stages addressed that forest fires occur very quickly and significant sensor data traffic congestion can occur during a single time unit. The first stage factor is $f_{\mathrm{risk}}$, derived from the forest fire prediction model, and the second stage factor was the number of packets in the node queue. Thus, ADX-MAC could adjust *T* when considering both the traffic situation and forest fire probability.

The proposed approach was verified experimentally by comparing it with X-MAC, and confirmed that ADX-MAC average throughput and energy efficiency were approximately 19% and 24% superior to X-MAC, respectively. Duty-cycle length changes with $f_{\mathrm{risk}}$ were also investigated, and *T* was shortened as forest fire probability increased, ensuring forest fires could be detected faster.

The proposed ADX-MAC protocol for forest fire detection is suitable for various natural disasters. Even if the forest fires are limited, ADX-MAC sensors could be incorporated into existing infrastructure to significantly reduce economic costs.

## Figures and Tables

**Table 1:** Fire damage data from the forest fire prediction model.

Field	Description	Example
Year_No	Annual number (starting from 1 every year)	1
Month_No	Annual number (starting from 1 every year)	1
F_Year	Year of occurrence	1991
F_Month	Month of occurrence	1
F_Day	Day of occurrence	13
F_Time	Time of occurrence	13:40
F_Week	Date of occurrence	SUN
E_Year	Year of extinguish	1991
E_Month	Month of extinguish	1
E_Day	Day of extinguish	14
E_Time	Completion time of extinguish	14:55
R_Time	Extinguish time	19:30
Loc_Type	Station 1 (with errors)	Ulsan
G_Offices	Station 2 (with errors)	Ulsan
Loc_SiDo	City (Si), State (Do)	Ulsan
Loc_Gung	Street 1 (Gun, Gu)	Buk
Loc_Dong	Street 2 (Dong)	
Loc_Ri	Street 3 (Lee)	
PNU_Nam	Other addresses	Mt. 79-1
Owner_Ty	Ownership type	
F_Type	Types of forest fires	
F_Cause (11 items)	Cigarette burning, trash incineration, building fire transfer, arson, field incineration, mistake by children, climbing, in operation, <rest>	Construction site, bonfire
D_Area	Damage area	14
D_AreaU	Additional damage area	0
D_AreaT	Total damage area	14
D_Amount	Amount of damage	1260
Temprt	Temperature	2.8
Wind_Dir	Wind direction	NNW
Wind_Sped	Wind speed	6
Humidity	Relative humidity	26
Lapse_Day	Elapsed days since 5 mm or more rainfall	6
R_Amount	Rainfall	1.8
Coord_X	X coordinate for location	416976.8
Coord_Y	Y coordinate for location	229115.2
Address	City (Si), State (Do) (Including special metropolitan city)	Ulsan Metropolitan City, Bukgu

**Table 2:** Correlation between forest fire frequency and climate factors.

Factor	Correlation $\left(w_{X}\right)$	Test Statistic
Temperature (*TP*)	−0.043	−0.7429
Wind speed (*WS*)	0.304	5.5086
Humidity (*H*)	−0.198	−3.4870
Rainfall (*R*)	−0.105	−1.8226

**Table 3:** Forest fire risk analysis for Mt. Gangneung, 2017.

Factor	January	February	March	April	May	June	July	August	September	October
**Measured climate factor**										
*WS* (m/s)	3	3.1	2.4	2.8	23	1.9	2	1.6	2.3	1.9
*H* (%)	43.3	38.2	48.5	44.3	29	61.4	67.9	74.4	61.2	67.2
*R* (mm)	48.5	3.5	48.4	39.8	0	27.2	238.1	444.1	45.7	114.5
*TP* (°C)	1.5	3.5	7.1	15.4	17.9	21.2	26.8	24.3	21.3	15.2
**Stair function test statistic**										
*WS* (m/s)	0	0	0	0	4	0	0	0	0	0
*H* (%)	2	3	2	2	4	0	0	0	0	0
*R* (mm)	4	4	4	4	4	4	2	0	4	3
*TP* (°C)	0	0	0	0	0	1	2	1	1	0
**Forest fire prediction model**										
$\;f_{\mathrm{risk}}$	0	0	0	0.0001	0.5610	0	0.0003	0.0003	0.0001	0.0001

**Table 4:** Risk Analysis of Forest Fire in Mt. Surak, 2017.

Factor	January	February	March	April	May	June	July	August	September	October
**Measured climate factor**										
*WS* (m/s)	2.3	2.5	2.4	2.6	2.4	5	2	2.3	2	1.9
*H* (%)	54.6	53.7	48.1	52.4	52.2	43	77.2	70.8	60.9	56.3
*R* (mm)	0	0	0	0	0	0	0	0	0	0
*TP* (°C)	−1.8	−0.2	6.3	13.9	19.5	23.3	26.9	25.9	22.1	16.4
**Stair function test statistic**										
*WS* (m/s)	0	0	0	0	0	1	0	0	0	0
*H* (%)	1	1	2	1	1	2	0	0	0	1
*R* (mm)	0	0	0	0	0	4	0	0	0	0
*TP* (°C)	0	0	0	0	0	1	2	2	1	0
**Forest fire prediction model**										
$\;f_{\mathrm{risk}}$	0.0006	0.0064	0.0469	0.0670	0.0806	1.102	0.0254	0.0385	0.0377	0.0332

**Table 5:** Network topology and simulation environment.

Parameter	Value	Parameter	Value
Bandwidth	250 Kbps	Node queue size	10
Sensing range	250 m	Packet (frame) size	50 bytes
Data transmit rate	5 ms	Transmit power	86.2 mW
Wake-up time	15 ms	Idle power	52.2 mW
ACK transmit time	1 ms	Receive power	96.6 mW
SP transmit time	3 ms	Sleep power	0.0183 mW
Initial duty-cycle length	100 ms		
